# Systematic Review of Calcineurin Inhibitors and Incidence of Skin Malignancies after Kidney Transplantation in Adult Patients: A Study of 309,551 Cases

**DOI:** 10.3390/curroncol30060430

**Published:** 2023-06-13

**Authors:** Aleksandra Kulbat, Karolina Richter, Tomasz Stefura, Marta Kołodziej-Rzepa, Michał Kisielewski, Tomasz Wojewoda, Wojciech M. Wysocki

**Affiliations:** 1 The Maria Sklodowska-Curie National Research Institute of Oncology, 02-781 Warsaw, Poland; alexandra.kulbat@gmail.com; 2 Department of General, Oncological and Vascular Surgery, 5th Military Clinical Hospital in Kraków, 30-901 Kraków, Poland; 3 Department of Medical Education, Jagiellonian University Medical College, 30-688 Kraków, Poland; 4 Chair of Surgery, Faculty of Medicine and Health Sciences, Andrzej Frycz Modrzewski Kraków University, 30-705 Kraków, Poland; karolinaa.richter@gmail.com

**Keywords:** calcineurin inhibitors, cyclosporine, tacrolimus, skin cancer, melanoma skin cancer, non-melanoma skin cancer, renal transplant recipients, renal transplantation, meta-analysis

## Abstract

The purpose of this systematic review and meta-analysis was to compare the risk of non-melanoma skin cancer (NMSC) and melanoma development in renal transplant recipients who receive calcineurin inhibitors to that of patients treated with other immunosuppressive agents, and investigate the possible association between the type of maintenance immunosuppression and the incidence of NSMC and melanoma in this group of patients. The authors searched databases such as PubMed, Scopus, and Web of Science for articles that would help establish the influence of calcineurin inhibitors on skin cancer development. The inclusion criteria for the study consisted of randomized clinical trials, cohort studies, and case-control studies that compared patients who received kidney transplants and were treated with a calcineurin inhibitor (CNI), such as cyclosporine A (CsA) or tacrolimus (Tac), to those who received alternative immunosuppressants and did not receive a CNI. Seven articles were analyzed overall. The results revealed a correlation between CNI treatment in renal transplant recipients and increased total skin cancer risk (OR 1.28; 95% CI: 0.10–16.28; *p* < 0.01), melanoma risk (OR 1.09; 95% CI: 0.25–4.74; *p* < 0.01), and NMSC risk (OR 1.16; 95% CI: 0.41–3.26; *p* < 0.01). In conclusion, the calcineurin inhibitors used after kidney transplantation are associated with a higher risk of skin cancer—both non-melanoma and melanoma—when compared with other immunosuppressive therapies. This finding suggests that careful monitoring for skin lesions in post-transplant patients must be conducted. However, the decision on the kind of immunotherapy used should always be considered on an individual basis for each renal transplant recipient.

## 1. Introduction

According to the World Health Organization (WHO), approximately 300,000 kidney transplants are performed each year worldwide. This number is constantly growing, reflecting the recognition of the benefits of kidney transplantation in patients with end-stage renal disease. The transplanted kidney takes over the function of the damaged kidneys and helps to restore the patient’s health, offering improved survival, better quality of life, and lower treatment costs compared with regular lifelong dialysis [1,2,3].

After a kidney transplant, long-term immunosuppression is necessary to prevent rejection of the transplanted kidney. The specific immunosuppressive regimen varies depending on the individual patients and their levels of immunological risk, but typically involves taking several oral drugs for the rest of the patient’s life [4]. The immunosuppressive drugs work by suppressing the kidney recipient’s immune system and preventing it from attacking the transplanted kidney [5].

Calcineurin inhibitors (CNIs), including CsA and Tac, are immunosuppressive drugs used extensively in the post-transplantation period to prevent the rejection of the transplanted organ [6,7]. These immunosuppressive agents inhibit the action of calcineurin, an enzyme that activates T-lymphocytes in the immune system, which plays a key role in cell-mediated immunity. However, the use of CNIs has been linked to an increased risk of developing various skin malignancies in renal transplant recipients due to continuous immunosuppression [6]. The increased risk of skin cancer in patients receiving CNI treatment is associated with several pathogenetic mechanisms that are often discussed. These mechanisms include the weakening of the immune system, which is a natural defense mechanism against tumor development and growth. The impact of immunosuppression on the skin can result in a decreased ability to protect against UV radiation and DNA damage. Additionally, calcineurin inhibitors can directly affect skin cells, leading to increased cell proliferation. This increased proliferation of skin cells can contribute to an elevated risk of developing skin cancer. Moreover, patients receiving immunosuppression are more susceptible to infections with oncogenic viruses, further increasing the risk of developing virus-related skin cancer [7,8]. Non-melanoma skin cancers (NMSC), including squamous cell carcinoma (SCC) and basal cell carcinoma (BCC), are the most common malignancies in renal transplant recipients [8,9]. An increased frequency of melanoma has also been reported among renal transplant recipients [8,9,10,11].

A limited number of studies have investigated the use of CNIs after kidney transplantation and the potential for skin malignancy development; therefore, we decided to systematically review the available evidence with the objective of comparing the carcinogenic potential of immunosuppressive regimens including CNIs with those that do not include CNIs. Additionally, we aimed to provide the number needed to harm (NNH) for skin malignancies in kidney transplant patients who need to take lifelong immunosuppressive drugs.

The principal aim of our meta-analysis was to compare the incidence of skin malignancies in patients who had undergone renal transplantation treated with CNIs to those treated with other immunosuppressive agents. 

To the best of our knowledge, this is the first systematic review and meta-analysis aimed at establishing a correlation between CNI use and the risk of skin malignancies in renal transplant recipients.

## 2. Materials and Methods

This study was designed to analyze the influence of CNIs, such as CsA and Tac, on the incidence of skin cancer after kidney transplantation. The study compared two therapeutic options used in renal transplant recipients: CNIs (CNIs group) and other immunosuppressants (no CNIs group). This systematic review and meta-analysis has been registered in PROSPERO under the unique identifier “CRD42023396207”. As a systematic review and meta-analysis, this study did not require any ethical approval or participant consent.

### 2.1. Search Terms

The authors followed the PRISMA guidelines to ensure the highest quality of their work [12]. The search was conducted using PubMed, Scopus, and Web of Science databases and was limited to studies published between March 1996 and November 2022. The search strategy was based on the following terms: “Cyclosporine”, “Cyclosporin”, “Ciclosporin”, “Tacrolimus”, “calcineurin inhibitors”, “CNI”, “skin neoplasms”, “skin cancers”, “skin cancer”, “squamous cell cancer”, “squamous cell carcinoma”, “squamous cell carcinomas”, “Squamous Cell Neoplasms”, “basal cell carcinoma”, “SCC”, “BCC”, “melanoma”, “kidney transplantation”, “renal transplantation”, “kidney transplant”, and “renal transplant”. All of the above-mentioned terms were combined using operators such as “AND” and “OR”. All found abstracts were screened for inclusion by two members of the review team (AK and KR) under the supervision of senior authors (TS, MKR, MK, TW, and WMW). The selected abstracts were thoroughly assessed and included in the meta-analysis based on the inclusion criteria. 

### 2.2. Eligibility Assessment

The authors conducted an eligibility assessment for all full-text articles that passed the abstract screening. We included randomized clinical trials, cohort studies, and case-control studies that reported on post-renal transplantation patients and compared the risk of developing melanoma (M) and non-melanoma skin cancer, such as squamous cell and basal cell carcinomas, between those treated with cyclosporine or tacrolimus and those who were treated with immunosuppressants other than CNIs (i.e., CNIs group vs. no CNIs group). We only analyzed articles that had full text and were written in English. The authors excluded all meta-analyses, systematic reviews, studies on pediatric kidney transplants, and otherwise irrelevant studies. 

### 2.3. Data Extraction

Two reviewers recorded relevant data from the selected papers. At least two authors extracted data from each article. In the event of conflicting data, one of the senior authors reviewed the study for further discussion and assistance with the decision regarding the inclusion of data.

### 2.4. Outcomes of Interest

The following data were extracted from the included studies: publication year, first author, title, type of study, country, follow-up period in years, patient sex, mean or median age of patients at the time of transplantation, transplant period (defined as the range of years during which the kidney transplantation was performed), median time from transplantation to skin malignancy diagnosis in years, sample size (defined as the population analyzed and separated into samples of kidney transplant recipients on CNI therapy and those without CNI therapy), type of skin malignancy diagnosed, including melanoma, non-melanoma skin cancer (NMSC), and total skin cancer in the CNIs and no CNIs groups. The authors adhered to the ethical guidelines and respected patient data ownership. The data used in this meta-analysis were obtained from publicly available literature and were de-identified to ensure patient confidentiality and privacy. Furthermore, the authors have made every effort to comply with the relevant copyright regulations and intellectual property rights while citing and referencing the original sources appropriately.

### 2.5. Quality Assessment

The quality of the included articles was evaluated using the Cochrane risk-of-bias tool for randomized trials (RoB 2) (The Cochrane Collaboration, 2020, London, UK). Several factors were recorded and measured, such as “Randomization process”, “Deviations from the intended interventions”, “Missing outcome data”, “Measurement of the outcome”, and “Selection of the reported result”. Each article was evaluated as “high risk”, “low risk”, or “some concerns”.

### 2.6. Statistical Analysis

Statistical analysis was carried out using R 4.2.2 (R Core Team, 2023) using publicly-available tidyverse and metabin packages. The authors divided the studies into three groups and analyzed the risk of melanoma, non-melanoma skin cancer, and total skin cancer. The relationship between CNI use and skin cancer risk was evaluated using the odds-ratio (as the effect size metric) and corresponding 95% confidence interval. Pooled estimates were obtained using the Mantel-Haenszel method [13,14,15]. The exact calculation was used for MSC due to presence of cells with zero frequency, as has been recommended [16]. For the heterogeneity variance calculation (tau-squared), the Der-Simonian Laird (1986) estimator was utilized. The Knapp–Hartung adjustment for the CI of the effect was also used, in order to account for between-study heterogeneity [17]. 

We assessed homogeneity between studies using the I-squared value and Q statistic. Due to low event/trial count, the 95% CI was also reported for the I-squared, as has been recommended [18]. We examined all of the included studies for sources of heterogeneity. As these populations are geographically diverse, the true risk difference may vary, and thus the random-effects model was selected to account for cross-population differences (aside from sampling error). 

## 3. Results

### 3.1. Article Selection

**Figure 1:**
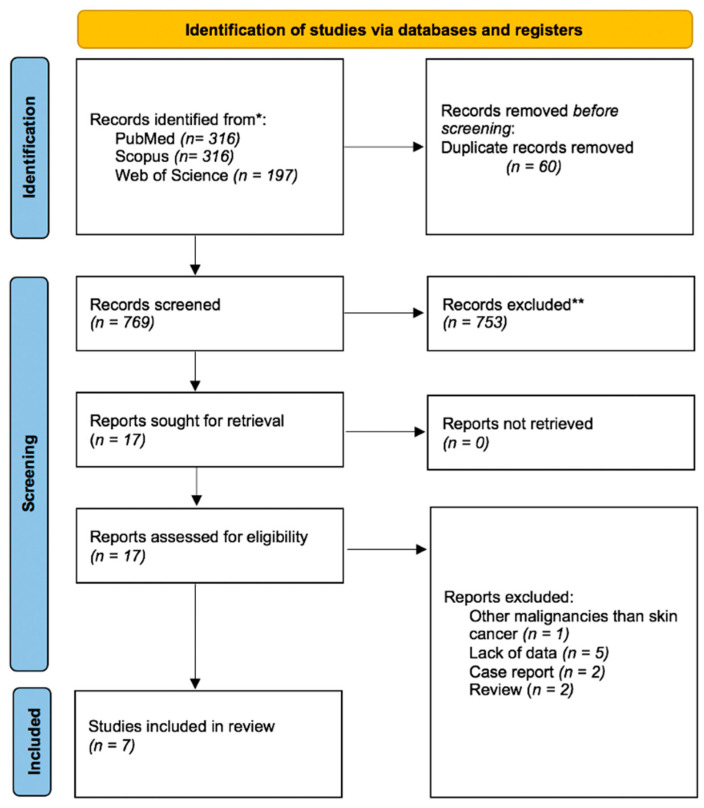
PRISMA flow diagram of the study inclusion process.

The primary search resulted in 829 records, and after the removal of duplicates, 769 articles were identified. Among these, 452 were not relevant to our study objective, 215 were case reports, 7 were on pediatric transplants, 59 were not available in English, 21 had no significant data, and 15 were reviews. After screening, 17 articles met the criteria for full-text review. In total, 10 studies were excluded by the authors after reanalysis due to the lack of relevant data. Finally, 7 papers that included a total of 309,551 patients were selected for the meta-analysis (Table 1). The eligibility screening process is depicted in detail in the PRISMA flow diagram (Figure 1).

### 3.2. Article Characteristics

Seven articles were included in the statistical analysis. The chosen articles contained 309,551 renal transplant recipients in Europe, Asia, Australia, and the USA between 1966 and 2016. We stratified data into three subgroups to assess the following outcome measures: “melanoma risk”, “non-melanoma skin cancer risk”, and “total skin cancer risk”.

### 3.3. Patient Characteristics

The authors selected and included seven articles, and a total of 309,551 patients were analyzed. There were 270,962 patients who were undergoing therapy using CNIs, such as CsA or Tac, and 38,313 patients who were treated using immunosuppressants other than CNIs. The entire dataset for the specified outcome measures included the following: 1427 patients for melanoma risk, 13,743 patients for non-melanoma skin cancer risk, and 14,618 patients for total skin cancer risk.

### 3.4. Melanoma Risk

**Figure 2:**
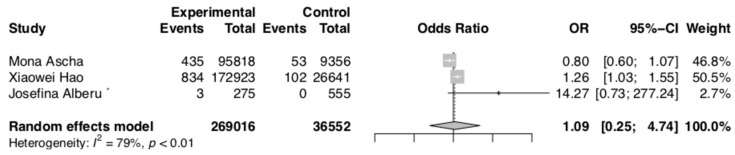
Melanoma risk [19,20,23].

The meta-analysis of outcomes presented by Alberu et al., Ascha et al., and Hao et al. [19,20,23] prove a statistically significant association between the use of CNIs and an increased risk of melanoma (OR 1.09; 95% CI: 0.25–4.74; *p* < 0.01). The results are presented in Figure 2. The NNH for melanoma risk and CNI treatment was 1541.1.

### 3.5. Non-Melanoma Skin Cancer Risk

**Figure 3:**
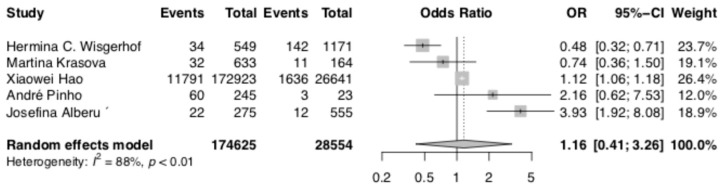
Non-melanoma skin cancer risk [19,21,23,24,25].

The analysis of non-melanoma skin cancer risk based on five studies (Alberu et al., Hao et al., Krasova et al., Pinho et al., and Wisgerhof et al. [19,21,23,24,25]) revealed a statistically significant difference with a lower risk of cancer development in the no CNIs group (OR 1.16; 95% CI: 0.41–3.26; *p* < 0.01). The data are shown in Figure 3. The NNH for non-melanoma cancer risk was 330.7. 

### 3.6. Total Skin Cancer Risk

**Figure 4:**
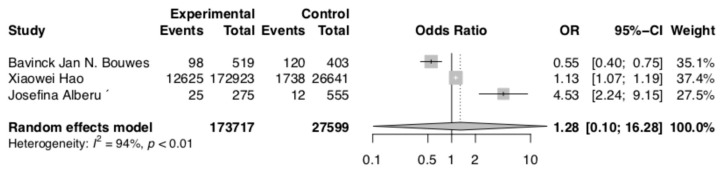
Total skin cancer risk [19,22,23].

Total skin cancer risk analysis included four articles (Alberu et al., Bouwes et al., Hao et al., [19,22,23]). Patients who were taking CNIs had higher total skin cancer risk (OR 1.28; 95% CI: 0.10–16.28; *p* < 0.01). The results are presented in Figure 4. The NNH for total cancer risk was 567.8.

### 3.7. Quality Assessment

**Figure 5:**
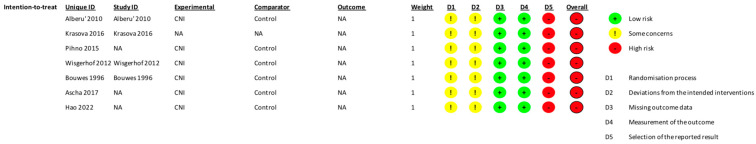
Quality assessment−Cochrane risk-of-bias tool for randomized trials (RoB 2) [19,20,21,22,23,24,25].

The risk of bias was low in certain aspects of the study, such as “Missing outcome data” and “Measurement of the outcome,” but there were some concerns in others, such as “Randomization process” and “Deviations from the intended interventions”. The risk of bias for “Selection of the reported result” was high. The overall quality of the included articles is evaluated in Figure 5.

## 4. Discussion

According to the “Renal Association Clinical Practice Guideline in Post-Operative Care in the Kidney Transplant Recipient”, CNI treatment should be started at the beginning of transplantation and continued until the graft is fully functional. Lifelong immunosuppressive therapy is required to prevent graft rejection and ensure proper functioning; however, studies have shown that CNI treatment can increase the risk of skin cancer due to the promotion of unrestricted cell division [26,27,28]. The potential mechanisms behind the increased risk of skin cancer with CNI therapy include the inhibition of DNA repair, the modification of immune function, and the suppression of the p53 protein [28]. Non-melanoma skin cancer, such as squamous cell carcinoma and basal cell carcinoma, is the most common type of skin malignancy affecting kidney transplant recipients [29,30,31,32]. 

We performed a statistical analysis on three parameters: melanoma risk, non-melanoma risk, and total cancer risk. All of them showed a statistically significant difference between a group of patients who were receiving CNIs and a group of patients who received alternative immunosuppressants and did not receive CNIs in the parameters studied. Melanoma risk was higher in the group of patients who were treated with CNIs (CsA and Tac) compared to the other group (OR 1.09; 95% CI: 0.25–4.74; *p* < 0.01). Non-melanoma skin cancer risk (OR 1.16; 95% CI: 0.41–3.26; *p* < 0.01) and total skin cancer risk (OR 1.28; 95% CI: 0.10–16.28; *p* < 0.01) were also slightly higher in the group of patients who had undergone renal transplantation treated with CNIs. Our findings, which are based on a large, pooled patient group of more than 300,000 kidney transplant recipients, indicate that permanent exposure to CNI treatment increases the risk of skin cancer. However, compared to other immunosuppressive drugs, this effect is not as significant as we initially expected. 

This association has been reported before; however, in contrast to previous studies [19,20,21,22,23,24,25], we were able to demonstrate statistically significant differences in all three parameters of melanoma risk, non-melanoma skin cancer risk, and total skin cancer risk. Our results suggest that the increased risk of skin cancer associated with CNI treatment may not be as widespread an issue as previously thought. In addition, we calculated the number needed to harm (NNH) parameter, which represents the number of patients that must be treated with CNIs for one individual to develop skin cancer [33]. We believe that this parameter most accurately illustrates the real risk related to CNI usage in this patient group in the context of skin cancer. The number needed to harm values were 1541.4 for melanoma risk, 330.7 for non-melanoma skin cancer risk, and 567.8 for total cancer risk. This means that every six hundredth patient taking CNI during the course of immunosuppression develops skin cancer (mainly basal and squamous cell carcinoma). Melanoma, with an NNH approaching 1541, is not a real threat to long-term CNI users. Our meta-analysis, which included more than 309,000 kidney transplant recipients across the included studies, is much larger than previous meta-analyses investigating the effect of CNIs on skin malignancies, and, to our knowledge, our study is the only one performed on a group of kidney recipients. This study takes into account research conducted on multiple continents and in different age groups, which reduces the influence of additional factors (such as sunlight exposure), as compared to previous studies. 

An NNH of 330 for non-melanoma skin malignancy associated with long-term immunotherapy using drugs from the group of CNIs leads to a very practical conclusion. This means that physicians dealing with kidney transplant recipients who are on long-term CNI treatment should carefully monitor patients for any signs of skin cancer and educate them on the importance of sun protection measures. Further research is needed to explore the underlying mechanisms of this association and to develop strategies to mitigate the risk of skin cancer in this patient population.

Our study has several important limitations. First, although a comprehensive literature search was conducted, the available literature on this topic is limited, and only a small number of relevant studies were found. Additionally, some of the studies included a variety of immunosuppressive drug regimens, making it difficult to isolate the effects of CNI monotherapy, and we were unable to include all data from the selected studies in our systematic review. Furthermore, some studies did not include and/or listed separately all types of skin malignancies (melanoma, squamous cell carcinoma, or basal cell carcinoma). Moreover, due to the variability in the nature of the included studies—such as differences in patient numbers, locations, and other additional factors which might increase the risk of skin cancers (such as high lifetime exposure to ultraviolet radiation from the sun, prior scars, chronic wounds, actinic keratosis, paler skin, Bowen’s disease, arsenic exposure, radiation therapy, tobacco smoking, poor immune system function, prior basal cell carcinoma, or HPV infection) [34,35]—there is a lack of uniformity that hinders the ability to draw strong conclusions from the systematic review. The selected studies encompassed a range of methodologies and characteristics, making it challenging to establish consistent patterns or generalize the findings. As a result, the diverse nature of the included studies limits the extent to which we can confidently interpret the results of the systematic review.

Another limitation of our study is the lack of specification regarding whether the patient is taking CsA or Tac, as well as the absence of information regarding other potential confounding factors; an example of this is the concurrent use of azathioprine, which has a higher potential carcinogenesis than CNIs [36]. This implies that the NNH estimates we have acquired must undergo verification by analyzing aggregated data obtained from transplant registries or alternative sources. It is evident that further measures are required to ensure their validity on a broader scale. 

Despite these limitations, the strength of our meta-analysis lies in our profound analysis of available relevant studies, strict inclusion criteria, and the integration of these studies to formulate a clinically important practical consensus. Due to the scarcity of studies examining the relationship between CNIs and skin cancer after kidney transplantation, the authors decided to provide the most up-to-date and complete conclusions based on our results and the available literature (see Section 5). To the best of our knowledge, the study is also unique and is the most precise meta-analysis that aims to establish a correlation between exposure to CNIs and the risk of skin cancer in renal transplant recipients. Another reason to study the potential relationship between CNIs and skin cancer is the increasing number of kidney transplant recipients and long-term survivors with lifetime CNI usage globally. Acknowledging the delayed consequences of therapy may be helpful in selecting therapy regimens and setting up necessary follow-up skin recommendations for patients on long-term CNI therapy.

Long-term immunosuppression in kidney transplant recipients has a beneficial effect on survival; however, it is associated with significant risks of skin cancer [37,38]. According to previous research, the risk of developing malignancies is four times higher in individuals who have undergone transplantation compared to the general population. Consequently, most international clinical guidelines recommend regular screening for skin, cervical, and colorectal cancer during the post-transplant period. It is therefore obligatory to inform patients and educate them on self-examination techniques and to encourage them to make frequent follow-up visits. Guidelines recommend that kidney transplant recipients should be screened for skin cancer occurrence at least twice a year for five years after transplantation [26,27,37,39,40,41,42]. Considering the potential for delayed development of skin malignancies in patients receiving calcineurin inhibitors (CNIs), we would recommend lifelong and continuous surveillance of the skin.

## 5. Conclusions

We showed that kidney transplant recipients undergoing long-term CNI therapy exhibit a statistically higher incidence of skin cancer compared to patients receiving alternative immunosuppressive treatments. However, the observed difference does not appear to have significant clinical implications when compared to immunosuppressive regimens that do not include CNIs. The risk of skin melanoma is also elevated, albeit to a considerably lesser extent, which may be disregarded in a clinical context. Early detection of non-melanoma skin cancers and melanoma is crucial for achieving favorable treatment outcomes. Detecting these cancers at an early stage allows for a more conservative therapeutic approach, minimizing the need for extensive interventions and improving long-term prognosis. Therefore, regular skin examinations and patient education on self-examination techniques are very important. It is also crucial to individualize the schedule for follow-up screenings based on risk factors and the need for a multidisciplinary approach involving dermatologists. We propose the implementation of lifelong skin monitoring sessions for patients with transplanted kidneys who are on long-term immunosuppression, including regimens with CNI. Furthermore, we strongly advocate for patient education regarding this undesired effect of CNIs to enhance awareness and facilitate early detection of skin lesions by the patients themselves. 

Additionally, in order to obtain a more comprehensive understanding of the topic, we recommend conducting further meta-analyses that take into account specific subgroups of patients receiving distinct immunosuppressive drugs. Unfortunately, this is currently unfeasible due to insufficient data in the available studies and challenges associated with data extraction. Our meta-analysis encountered numerous limitations and obstacles, and the estimated outcomes and assessments should be verified through the analysis of aggregated data from transplant registries or other accessible sources. It is evident that additional efforts are required to ensure the validity and objectivity of the findings on a larger scale.

## Figures and Tables

**Table 1:** Characteristics of included studies.

Publication Year	First Author	Title	Study Design (Rct/Cohort /Case-Control)	Country	Sample (n)	Follow-Up in Years	Sex (% Women)	Mean Or Median Age Of Transplantation	Transplant Period	Sample on CNI Therapy (n)	Sample Without CNI Therapy (n)	Sample CNI-MSC (n)	Sample CNI-NMSC (n)	Sample CNI-Total Skin Cancer (n)	Sample No CNI-MSC (n)	Sample No CNI-NMSC (n)	Sample No CNI-Total Skin Cancer (n)	Median Time to Diagnosis in Years
2022	Xiaowei Hao [19]	Skin cancer outcomes and risk factors in renal transplant recipients: Analysis of organ procurement and transplantation network data from 2000 to 2021	Cohort	China	199,564	7.23	39.45	49.49	2000–2014	172,923	26,641	834 (0.48%)	11,791 (6.82%)	12,625 (7.3%)	102 (0.38%)	1636 (6.14%)	1738 (6.52%)	5.84
2017	Mona Ascha [20]	Risk Factors for Melanoma in Renal Transplant Recipients	Cohort	USA	105,174	NS	49.3	49.6	2004–2012	95,818	9356	435 (0.45%)	x	x	53 (0.57%)	x	x	NS
2016	Martina Krasova [21]	Immunosuppressive therapy in the posttransplant period and skin cancer	Cohort	Czech Republic	797	5.3	34.3	48.73	1980–2016	633	164	x	32 (5.06%)	x	x	11 (6.7%)	x	7.97
1996	Bavinck Jan N. Bouwes [22]	The risk of skin cancer in renal transplant recipients in Queensland, Australia. A follow-up study	Cohort	Australia	1098	11.7	62.4	45.9	1969–1994	519	403	x	x	98 (18.89%)	x	x	120 (29.78%)	4.7
2010	Josefina Alberú [23]	Lower Malignancy Rates in Renal Allograft Recipients Converted to Sirolimus-Based, Calcineurin Inhibitor-Free Immunotherapy	RCT	International	830	3.1	29.5	42.6	NS	275	555	3 (1.1%)	22 (8%)	25 (9.1%)	0 (0%)	12 (2.16%)	12 (2.16%)	NS
2015	André Pinho [24]	Non-melanoma skin cancer in Portuguese kidney transplant recipients—incidence and risk factors	Case control	Portugal	288	3.67	34	47	2004–2013	245	23	x	60 (24.49%)	x	x	3 (13.04%)	x	5.35
2012	Hermina C. Wisgerhof [25]	Kidney Transplant Recipients with Cutaneous Squamous Cell Carcinoma Have an Increased Risk of Internal Malignancy	Cohort	Netherlands	1800	11	38	43	1966–2006	549	1171	x	34 (6.19%)	x	x	142 (12.13%)	x	19.4

Abbreviations: CNI, calcineurin inhibitor; MSC, melanoma skin cancer; NMSC, non-melanoma skin cancer; NS, not stated; RCT, randomized controlled trial.
